# Eventos Cardiovasculares Evitáveis: Um Sério Efeito Colateral da Pandemia de COVID-19

**DOI:** 10.36660/abc.20210113

**Published:** 2021-03-03

**Affiliations:** 1 Hospital Israelita Albert Einstein São PauloSP Brasil Hospital Israelita Albert Einstein, São Paulo, SP – Brasil

**Keywords:** COVID-19, Betacoronavírus, Pandemia, Infarto do Miocárdio, Insuficiência Cardíaca, Doenças Cardiovasculares/mortalidade, Vacinação

Logo após o início da pandemia de COVID-19, um sentimento geral surgiu entre os médicos: muitos pacientes teriam desfechos desfavoráveis devido à demora na procura por serviços médicos por medo de contaminação. Pacientes com infarto agudo do miocárdio, insuficiência cardíaca descompensada ou acidente vascular cerebral (AVC) permaneceriam em casa ou iriam para o hospital tardiamente. A investigação de doenças possivelmente graves como o câncer seria adiada. Além disso, o colapso do sistema de saúde seria um fator contribuinte para o cuidado inadequado de doenças outras que não a COVID-19.

Levou alguns meses para que os pesquisadores coletassem dados sobre as nuances desse fenômeno. Diversas publicações têm confirmado as expectativas, lançando luz sobre um aspecto que deve ser discutido e divulgado.

Nesse contexto, este número da ABC publica uma análise do impacto dos primeiros meses da pandemia sobre o número de procedimentos médicos, internações e óbitos intra-hospitalares por doenças cardiovasculares no âmbito do serviço público de saúde brasileiro.^1^ Os números esperados para março a maio de 2020, baseados nas tendências de 2016 a 2019, foram confrontados com os números observados durante a pandemia. Os principais resultados indicam que o número de procedimentos, cirurgias, internações e óbitos hospitalares foi muito inferior ao esperado. Os autores descrevem uma redução de 15% nas internações por doenças cardiovasculares. Os óbitos hospitalares por doenças cardiovasculares também diminuíram, mas não na mesma proporção (-8%). Como consequência, a letalidade intra-hospitalar aumentou 9%.^1^

O número reduzido de procedimentos cardíacos e internações hospitalares devido a urgências cardiovasculares é compatível com outros relatos do Brasil e de outros países.^2^^–^^4^ Essa observação, por si só, não seria um problema se desfechos relevantes como óbitos, insuficiência cardíaca pós-infarto do miocárdio e incapacidades pós-AVC não tivessem aumentado. Infelizmente, não é o caso. Brant et al.^5^ publicaram uma análise abrangente do excesso de mortalidade cardiovascular de março a maio de 2020 nas seis capitais brasileiras com maior número de óbitos por COVID-19. Os autores encontraram um excesso no total de óbitos cardiovasculares na maioria das cidades, principalmente devido a causas cardiovasculares não especificadas, que foram correlacionadas com um aumento nos óbitos domiciliares.^5^

As questões relacionadas às consequências indiretas e nocivas da pandemia não se restringem às doenças cardiovasculares. Várias publicações têm destacado demanda reduzida por serviços oncológicos e menor número de exames diagnósticos e procedimentos terapêuticos para o câncer durante a pandemia.^6^^–^^9^

Portanto, os danos colaterais da pandemia de COVID-19 são claros e alarmantes, e estratégias para minimizar eventos evitáveis devem ser desenvolvidas e implementadas. Isto é especialmente verdadeiro no contexto das doenças cardiovasculares, principal causa de óbitos no mundo e no Brasil.^10^

Obviamente, a solução ideal é controlar a própria pandemia, o que leva tempo e dependerá do sucesso do programa de vacinação, que está sempre ameaçado pelo surgimento de novas variantes do coronavírus da síndrome respiratória aguda grave 2 (SARS-CoV-2). Enquanto a transmissão comunitária do vírus estiver em níveis elevados, outras iniciativas podem ajudar.

Em primeiro lugar, é obrigatório alertar os médicos e pacientes para o perigo de delongas no diagnóstico e no tratamento de doenças cardiovasculares, malignidades e outros processos patológicos. Os médicos devem dominar a tarefa frequentemente difícil de equilibrar os benefícios e riscos de encaminhar pacientes para procedimentos diagnósticos e internações hospitalares. O correto discernimento sobre a urgência depende de um misto de educação continuada, capacitação e bom senso. Campanhas públicas voltadas para leigos também podem ser úteis.

Em segundo lugar, os serviços de saúde devem estar preparados para minimizar os riscos aos pacientes e profissionais de saúde durante a pandemia. As sociedades médicas têm o papel de orientar os profissionais de saúde para a realização de procedimentos com segurança, como a Sociedade Brasileira de Cardiologia tem feito por meio da emissão de documentos com recomendações específicas.^11^^–^^13^ Hospitais de referência e ensino também podem contribuir compartilhando suas experiências e protocolos que podem ser utilizados por centros menores.^14^

Em terceiro lugar, devemos tirar proveito do atendimento de saúde remoto para preencher parcialmente a lacuna de acesso restrito à assistência médica nestes tempos de pandemia. Uso inteligente da telemedicina,^15^ visitas da equipe de saúde e coleta de sangue em domicílio, bem como teletransmissão do eletrocardiograma são alguns exemplos de práticas que podem ser adotadas.

Em conclusão, agora está claro que os impactos da pandemia de COVID-19 vão muito além dos óbitos por pneumonia viral e aspectos socioeconômicos. Os pacientes estão mais relutantes e têm adiado investigações médicas, em grande parte devido ao medo de serem infectados pelo SARS-CoV-2. Embora compreensíveis do ponto de vista do paciente, essas atitudes apresentam riscos elevados, principalmente quando se trata de doenças cardiovasculares e câncer. Esforços devem ser feitos para limitar os eventos evitáveis e para minimizar as consequências trágicas dessa pandemia em curso ou outras tragédias que possam nos atingir futuramente.
