# Supplementary material for: Eventos Cardiovasculares Evitáveis: Um Sério Efeito Colateral da Pandemia de COVID-19
Source: Arq Bras Cardiol. 2021 Mar 3;116(3):381–2. [Article in Portuguese] doi: 10.36660/abc.20210113 (PMC8159556; doi:10.36660/abc.20210113)
Supplement: Translation

# Translation: Avoidable Cardiovascular Events: A Serious Side Effect of the COVID-19 Pandemic

**Affiliations:** 1 Hospital Israelita Albert Einstein São PauloSP Brazil Hospital Israelita Albert Einstein, São Paulo, SP – Brazil

**Keywords:** COVID-19, Betacoronavirus, Pandemics, Myocardial Infarction, Heart Failure, Cardiovascular Diseases/mortality, Vaccination

Soon after the beginning of the COVID-19 pandemic, a general feeling arose among the doctors: many patients would have poor outcomes due to delays in seeking medical services for fear of infection. Patients with acute myocardial infarction, decompensated heart failure or stroke would stay at home or go to the hospital late in the course of the event. Investigation of potentially serious diseases, such as cancer, would be postponed. Besides, healthcare collapse would prove to be a contributor to inadequate care of non-COVID-19 diseases.

It took some months for the researchers to gather data on the nuances of this phenomenon. Several publications have confirmed the expectations, shedding light on an aspect that must be discussed and disseminated.

In this context, this issue of ABC publishes an analysis of the impact of the first months of the pandemic on the number of medical procedures, hospital admissions, and in-hospital deaths due to cardiovascular diseases in the setting of the Brazilian public healthcare service.^1^ The numbers expected for March to May 2020, based on the trends from 2016 to 2019, were confronted with the numbers observed during the pandemic. The main results indicate that the number of procedures, surgeries, hospital admissions, and in-hospital deaths was much lower than expected. The authors describe a 15% decrease in hospital admissions due to cardiovascular diseases. In-hospital deaths due to cardiovascular diseases also dropped, but not to the same extent (-8%). As a consequence, in-hospital mortality increased by 9%.^1^

The reduced number of cardiac procedures and hospital admissions due to cardiovascular urgencies is consistent with other reports from Brazil and other countries.^2^^–^^4^ This observation, per se, would not be a problem if relevant outcomes, such as deaths, post-myocardial infarction heart failure and post-stroke disabilities had not increased. Unfortunately, this is not the case. Brant et al.^5^ have published a comprehensive analysis of excess cardiovascular mortality from March to May 2020 in the six Brazilian capitals with the highest number of COVID-19 fatalities. The authors found an excess in total cardiovascular deaths in most cities, especially due to unspecified cardiovascular causes, which were correlated with an increase in home deaths.^5^

Concerns about indirect, harmful consequences of the pandemic are not restricted to cardiovascular diseases. Several publications have highlighted a reduced demand for cancer services, and a lower number of diagnostic exams and therapeutic procedures for cancer during the pandemic.^6^^–^^9^

Therefore, the collateral damage of the COVID-19 pandemic is clear and alarming, and strategies to minimize avoidable events must be developed and implemented. This is especially true in the setting of cardiovascular diseases, the leading cause of death in the world and in Brazil.^10^

Obviously, the ideal solution is to control the pandemic itself, which takes time and will depend on the success of the vaccination program, which is always threatened by the emergence of new variants of the severe acute respiratory syndrome-related coronavirus 2 (SARS-CoV-2). As long as the community transmission of the virus is at high levels, other initiatives may help.

Firstly, it is mandatory to make doctors and patients aware of the danger of delaying diagnosis and treatment of cardiovascular diseases, malignancies, and other pathological processes. Physicians should master the frequently hard task of balancing the benefits and risks of referring patients to diagnostic procedures and hospital admissions. The correct discernment on the urgency depends on a mixture of continuing education, training, and common sense. Public campaigns targeting lay people may also be useful.

Secondly, healthcare services must be prepared to minimize risks to patients and healthcare professionals during the pandemic. Medical societies have a role in guiding the health providers to safely perform procedures, such as the Brazilian Society of Cardiology has done by issuing documents with specific recommendations.^11^^–^^13^ Teaching, reference hospitals can also contribute by sharing their experience and protocols that may be used by smaller centers.^14^

Thirdly, we should take advantage of remote healthcare to partially fill the gap of restricted access to medical assistance in these pandemic times. Intelligent use of telemedicine,^15^ home visits by the health team, blood collection at home, and electrocardiogram teletransmission are some examples of practices that may be performed.

In conclusion, it is now clear that the impacts of the COVID-19 pandemic go far beyond deaths due to viral pneumonia and socio-economic aspects. Patients are more reluctant and have postponed medical investigations, largely due to the fear of being infected with SARS-CoV-2. Although understandable from the patient’s perspective, these attitudes pose high risks, especially when it comes to cardiovascular diseases and cancer. Efforts must be made to limit preventable events and to minimize the tragic consequences of this ongoing pandemic or other disasters that might affect us in the future.
